# Bibliometric insights in advances of papillary thyroid microcarcinoma: Research situation, hot points, and global trends

**DOI:** 10.3389/fendo.2022.949993

**Published:** 2022-08-08

**Authors:** Kefan Chen, Zhongqing Wang, Wei Sun, Dalin Zhang, Ting Zhang, Liang He, Hao Zhang

**Affiliations:** ^1^ Department of Thyroid Surgery, the First Hospital of China Medical University, Shenyang, China; ^2^ Department of Information center, the First Hospital of China Medical University, Shenyang, China

**Keywords:** bibliometric, papillary thyroid microcarcinoma, visualized maps, research frontiers, management

## Abstract

**Background:**

Thyroid cancer has been on the rise over the last decade. Papillary thyroid microcarcinoma (PTMC) accounts for more than half of all thyroid cancers. Micropapillary carcinoma of the thyroid is a common but non-fatal form of thyroid cancer. To better comprehend, nearly two decades of scientific outputs were analyzed and summarized using bibliometric methods in this study.

**Methods:**

Approximately 1098 publications from 2000 and 2021 were included in WoS database through systematic retrieval. The general information was characterized, and developmental skeleton and research frontiers were explored. CiteSpace, VOSviewer, and R, Tableau were used to evaluate and visualize the results.

**Results:**

A total of 1098 publications from across 75 countries were identified. The annual number of publications showed an increasing trend in the past 21 years. China, Korea, the United States of America (USA), Italy, and Japan made remarkable contributions to the research of PTMC. Thyroid was the most productive journal. Miyauchi Akira published maximum articles. The utmost productive institution was the University of Ulsan. Risk stratification, active surveillance, and thermal ablation garnered the attention of researchers leading to novel approaches in the clinical diagnosis and treatment of micropapillary thyroid carcinoma.

**Conclusions:**

This bibliometric study provides a comprehensive analysis of global productivity, collaboration, and research hotspots within PTMC field, which will aid in directing research toward PTMC in the coming years.

## Introduction

Thyroid cancer is the most common endocrine tumor globally (1). In 2015, 3.2 million people developed thyroid cancer worldwide, resulting in 31,900 deaths (2, 3). Thyroid cancer ranked ninth in incidence in 2020, with 586,000 cases worldwide (4), a slight increase from 567,000 new cases in 2018 (1), rendering it a substantial public health problem universally. Papillary thyroid microcarcinoma (PTMC) is the most common thyroid cancer in clinical practice, defined as PTC involving tumors <1 cm in diameter. As a result, PTMC predominates in the context of diagnosis and treatment of thyroid cancer. An increasing number of people are being diagnosed with PTMC with ultrasound technology advancement. Many patients come to the clinic because of thyroid nodules, which can be detected in the physical examination, which help in their diagnosis (5). Cho et al. summarized the clinicopathological and prognostic changes of thyroid cancer in South Korea over the past 40 years. They found that the proportion of PTMC increased from 6.1% in 1962 to about 9% in 1990, to 54% in 2005, and 43.1% in 2009 (6), but there was no significant rise in mortality (7).

Even though many systematic reviews and meta-analysis have thoroughly and explicitly addressed PTMC research, only a few summarized this topic from the perspective of bibliometric analysis or provided the developing trends in this domain. Some studies performed bibliometric analysis but did not provide future trends for PTMC. Therefore, we aimed to conduct a bibliometric network analysis, which would offer an objective measurement in scientific literature and aggregate the opinions of multiple PTMC researchers.

As there was no comparable bibliometric analysis in the research field of PTMC, we applied related tools to systematically and objectively evaluate the research foundation, frontiers, and focus on PTMC.

## Methods

### Data collection

The data were retrieved from the Web of Science (WoS) Core Collection database using an advanced search strategy on January 16, 2022. The key topic for retrieval was TS=(“papillary microcarcinoma of the thyroid” or “papillary thyroid microcarcinoma” or “thyroid papillary micro-carcinoma” or “papillary microcarcinoma” or “small papillary thyroid carcinoma” or “thyroid micropapillary carcinoma”). The refining method was (1) document type (article and review); (2) language (English) (3); timespan: January 2000 – December 2021. Subsequently, 1098 publications were retrieved that comprised the final literature database set. Each document record included the title, author, keywords, abstract, year, organization, citation, and other relevant information. Since the data were retrieved from a public open-accessed database, ethical permissions were not required.

### Data analysis

This study adopted VOSviewer 1.6.17 and CiteSpace 5.8R3 for scientometric analysis, map cooperation networks (authors, institutions, and countries), keyword co-occurrence, and document co-citation clustering. The visualization of these collaborative networks in a discipline was based on the theory of co-citation, which posits that two documents share a co-citation correlation when cited together in another document. Rstudio v1.4.1717 was used to extract various data and draw statistical images for intuitive understanding. Tableau v10.5.0 was used to draw the world map, which represented the number of publications of various countries and regions.

## Results

### Publication year

**Figure 1:**
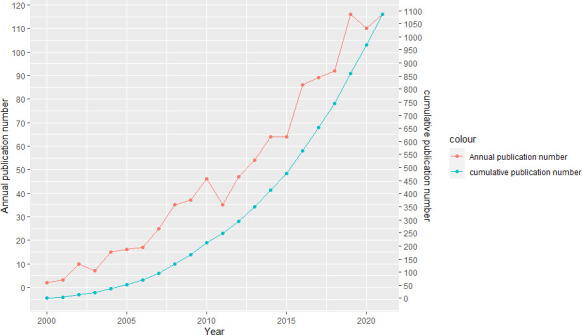
Publications of publications in PTMC research from 2000 to 2021. The red curve represents the annual publication number and the green curve represents the cumulative publication number.

According to WoS database search results, 1098 publications and reviews related to PTC were published between January 2000 and December 2021. The number of publications generally increased each year, indicating that PTMC research field gained attention gradually. Specifically, 121 publications were published in 2019, demonstrating the highest publication frequency. In 2000 and 2001, the publication frequency was the lowest, with two and three publications, respectively (**Figure 1**).

### Leading countries and institutions

**Figure 2:**
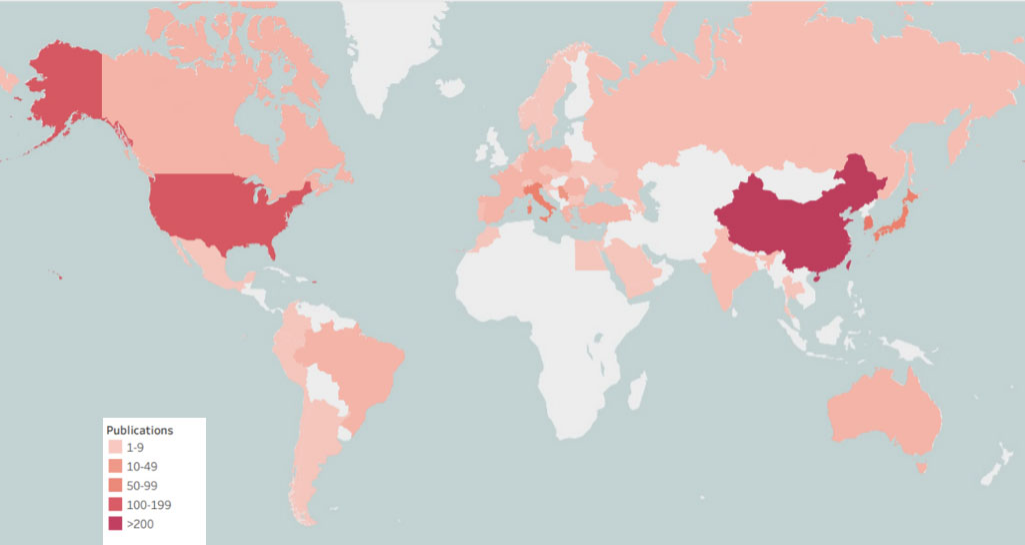
Global geographical distribution of PTMC publications.

**Figure 3:**
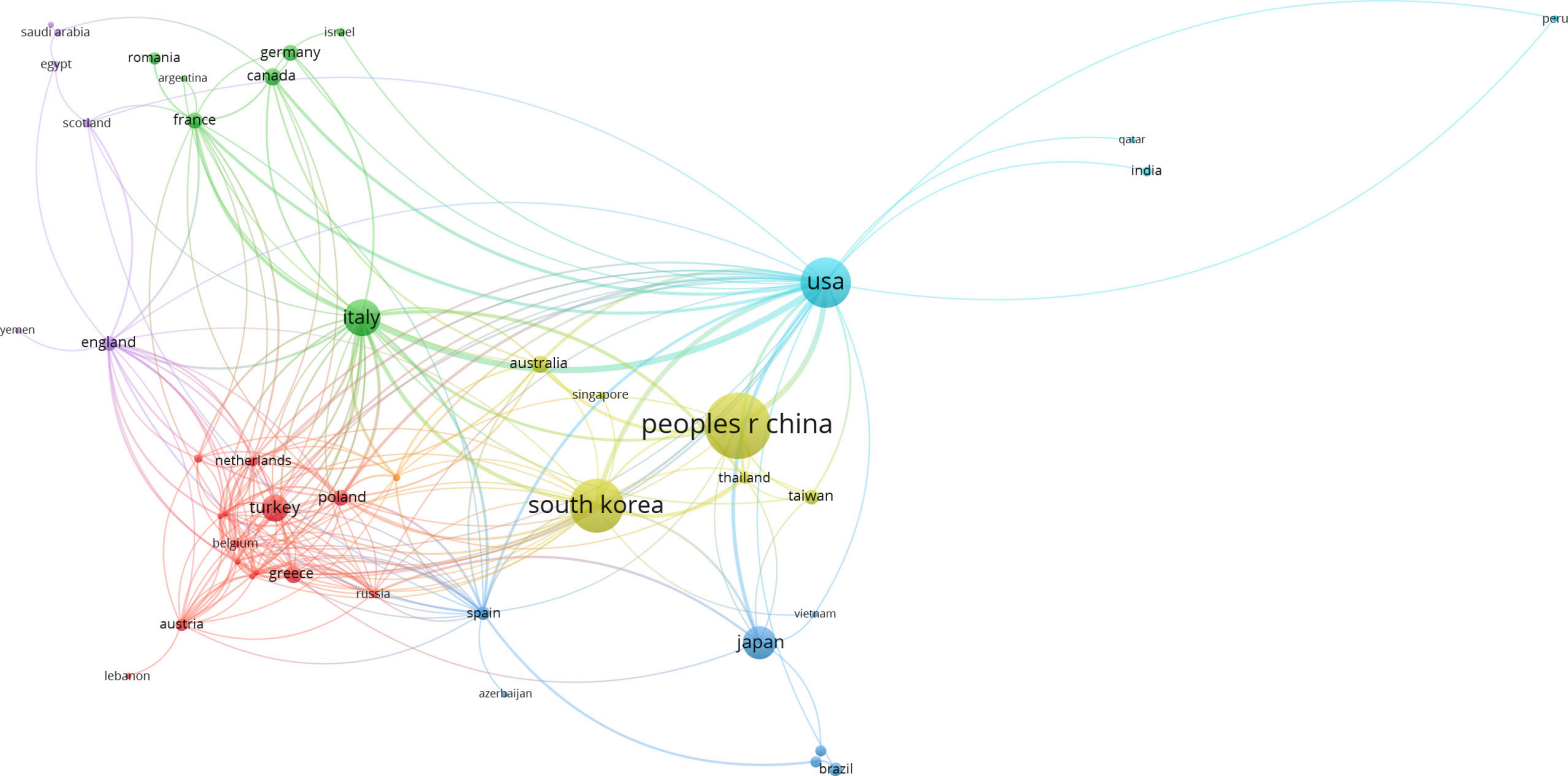
The international collaboration relationship networks. Bar length reflects the numerical size of each parameter. Dots represent country and larger dots indicate a larger number of publications of country. The clusters are labeled using different colors and the links represent citations or co-authorships of country, respectively.

**Figure 4:**
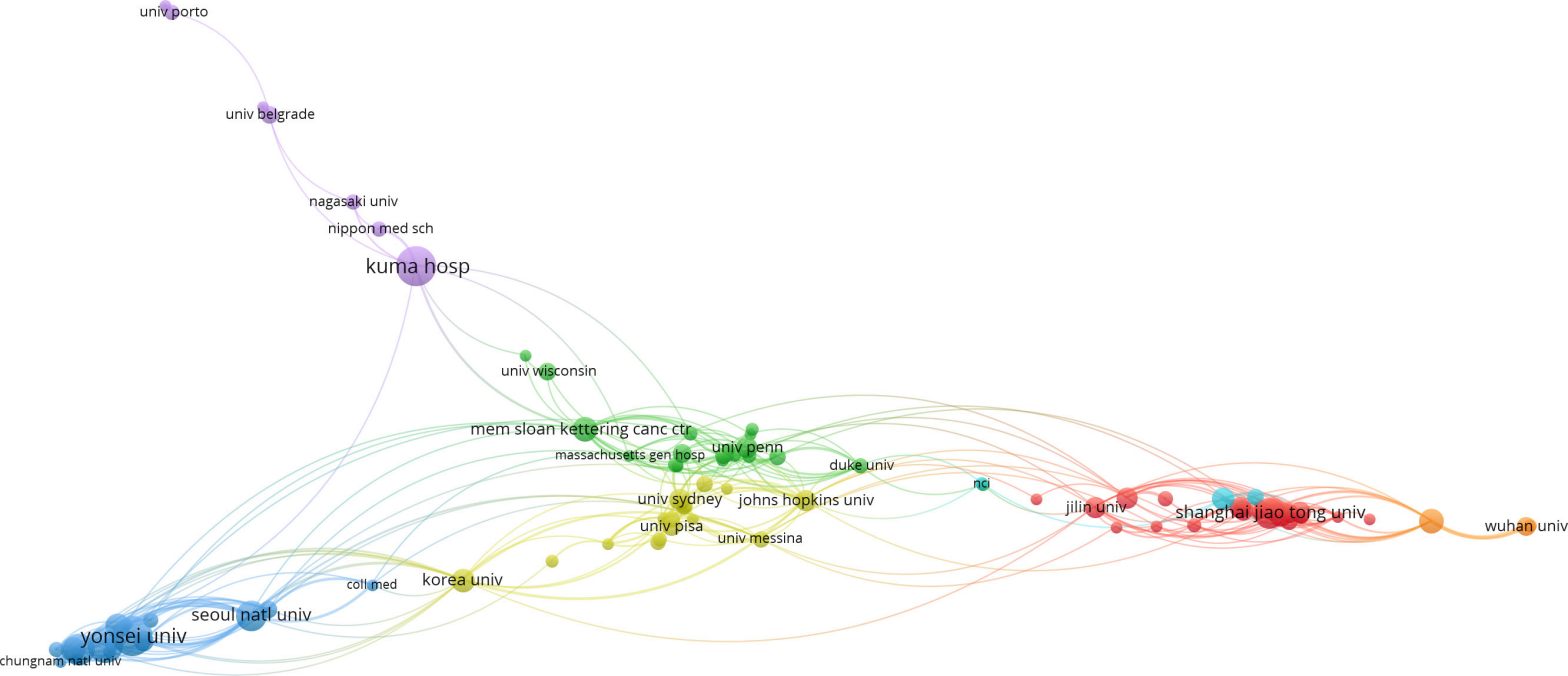
The visualization of co-authorships networks of the top 99 most productive institutions in PTMC field.

A world map was drawn to intuitively understand the total number of publications from 75 countries worldwide (**Figure 2**). North America, East Asia, and Western Europe reached remarkable achievements. China participated in the maximum number of studies; however, Korea’s H, G, and M indexes were the highest, signifying that the research results in Korea were of high quality (**Table 1**). Furthermore, intensive cooperation was observed among these countries (**Figure 3**). Among the top 10 research institutions globally, six were from South Korea, three from China, and one from Japan (**Table 2**). The University of Ulsan was the world’s most productive research institute regarding PTMC research (**Table 2** and **Figure 4**). The top 10 institutions published 17.28% of the papers.

**Table 1:** Top countries and institutions in PTMC field.

Country	Publications	Proportion of publications	Citations	Citations per publication	H-index	G-index	M-index
CHINA	329	29.99%	4627	14.06	34	67	1.70
KOREA	201	18.32%	5992	29.81	44	75	2.44
USA	152	13.86%	6164	40.55	43	84	2.05
ITALY	78	7.11%	3462	44.38	33	65	1.57
JAPAN	73	6.65%	4943	67.71	35	72	1.75
TURKEY	49	4.47%	399	8.14	11	18	0.65
GERMANY	18	1.64%	495	27.50	11	18	0.52
GREECE	18	1.64%	501	27.83	12	17	0.57
CANADA	17	1.55%	271	15.94	11	18	0.52
FRANCE	15	1.37%	407	27.13	12	16	0.57

H-index is Hirsch index; M-index is derived by dividing the h-index by career duration. G-index focuses on the cumulative contribution of paper citation frequency.

**Table 2:** The top 10 most productive institutions within PTMC research from 2000 to 2021 cumPubPerc= Cumulative publication percentage.

	Institute	Publication	cumPubPerc (%)	Country
1	University of Ulsan	101	2.96	KOREA
2	Yonsei University	95	5.74	KOREA
3	Seoul National University	92	8.44	KOREA
4	Kuma Hospital	67	10.40	JAPAN
5	Sungkyunkwan University	57	12.07	KOREA
6	Shanghai Jiao Tong University	43	13.33	CHINA
7	Inje University	39	14.47	KOREA
8	Korea University	33	15.44	KOREA
9	Fudan University	32	16.37	CHINA
10	Huazhong University of Science and Technology	31	17.28	CHINA

### Most active journals and highly cited publications

Academic journals serve as a medium for exchanging and disseminating knowledge in various disciplines. According to data analysis, 296 journals published in PTMC field. The 15 core journals were identified as per Bradford’s law, with focus given to the top ten journals (**Table 3**): Thyroid, World Journal of Surgery, Annals of Surgical Oncology, Endocrine, Journal of Clinical Endocrinology & Metabolism, Surgery, Endocrine Pathology, Medicine, Gland Surgery and Frontiers in Endocrinology. The quartile and categories were retrieved from Journal Citation Reports (JCR). Most listed journals were categorized into “endocrinology and metabolism” and “surgery.” Four of the ten magazines located at JCR quartile one were rated as high-quality. The h-index, g-index, and m-index of Thyroid, World Journal of Surgery, and Annals of Surgical Oncology had higher scores than other journals (**Table 3**). The journals mentioned above were frequently referred to by researchers.

**Table 3:** The core journals that published publications in PTMC field.

	NP		TC		H-index		G-index		M-index		JCR quartile		Categories		
THYROID	71		4202	35		64		1.75		Q1			SURGERY		
WORLD JOURNAL OF SURGERY	41		2718	28		41		1.47		Q2			ENDOCRINOLOGY & METABOLISM; PATHOLOGY		
ANNALS OF SURGICAL ONCOLOGY	30		1347	20		30		1.18		Q1			ENDOCRINOLOGY & METABOLISM		
ENDOCRINE	20		331	11		18		0.92		Q3			SURGERY		
JOURNAL OF CLINICAL ENDOCRINOLOGY & METABOLISM	20		2135	16		20		0.76		Q1			ENDOCRINOLOGY & METABOLISM		
SURGERY	20		1280	16		20		0.76		Q1			ENDOCRINOLOGY & METABOLISM		
ENDOCRINE PATHOLOGY	18		467	13		18		0.62		Q2			ONCOLOGY; SURGERY		
MEDICINE	18		143	8		10		1.00		Q3			ENDOCRINOLOGY & METABOLISM		
GLAND SURGERY	16		121	6		10		0.86		Q2			SURGERY		
FRONTIERS IN ENDOCRINOLOGY	14		87	7		8		1.40		Q1			ENDOCRINOLOGY & METABOLISM		

NP, Number of published; TC, Total citations.

The characteristics, dates, and countries of highly cited publications are listed in **Table 4**. Four of the publications were from the USA, three from Japan, and three from China, South Korea, and Italy. The most frequently cited publication was “Risk of malignancy in nonpalpable thyroid nodules: predictive value of ultrasound and color-doppler features” with 882 citations. Three of the ten publications explored the practice of ultrasound in the diagnosis of thyroid cancer, discussed the stage and prospect of Thyroid Imaging Reporting and Data System(TI-RADS), the capability of ultrasound in detecting benign and malignant nodules, and the suggestions for treatment (8–10). Two Japanese publications suggested that non-progressive PTMC should be monitored and treated, particularly in the elderly over 60, even with a low probability of disease progression. Three publications focused on the clinical treatment strategy of PTMC, including that PTMC and PTC should be treated with the same treatment strategy, and PTMC patients with obvious lymphadenopathy should be treated with therapeutic lymph node dissection. Prophylactic lymphadenectomy was not helpful in patients without obvious lymphadenopathy. For most patients with PTC, neither extensive surgery nor radioactive ablation reduced the recurrence rate compared to unilateral lobectomy (11–13). An American publication examined the causes of the increased incidence of thyroid cancer concerning electromagnetic radiation, obesity, height, smoking, etc. This study looked into the issue of overtreatment of thyroid cancer and emphasized the importance of patient preference and patient-centered decision-making (14).

**Table 4:** The characteristics of highly cited and the most impact classic publications in PTMC field.

	Publication	Citations	Journal	IF2020	Published year	Country
1	Risk of malignancy in nonpalpable thyroid nodules: Predictive value of ultrasound and color-Doppler features	882	JOURNAL OF CLINICAL ENDOCRINOLOGY & METABOLISM	5.958	2002	Italy
2	ACR Thyroid Imaging, Reporting and Data System (TI-RADS): White Paper of the ACR TI-RADS Committee	662	JOURNAL OF THE AMERICAN COLLEGE OF RADIOLOGY	5.532	2017	USA
3	Lymph node metastasis from 259 papillary thyroid microcarcinomas - Frequency, pattern of occurrence and recurrence, and optimal strategy for neck dissection	577	ANNALS OF SURGERY	12.969	2003	Japan
4	Papillary microcarcinoma of the thyroid - Prognostic significance of lymph node metastasis and multifocality	508	CANCER	6.86	2003	China
5	An observation trial without surgical treatment in patients with papillary microcarcinoma of the thyroid	503	THYROID	6.568	2003	Japan
6	Ultrasonography Diagnosis and Imaging-Based Management of Thyroid Nodules: Revised Korean Society of Thyroid Radiology Consensus Statement and Recommendations	430	KOREAN JOURNAL OF RADIOLOGY	3.5	2016	Korea
7	The Prognostic Significance of Nodal Metastases from Papillary Thyroid Carcinoma Can Be Stratified Based on the Size and Number of Metastatic Lymph Nodes, as Well as the Presence of Extranodal Extension	425	THYROID	6.568	2012	USA
8	Patient Age Is Significantly Related to the Progression of Papillary Microcarcinoma of the Thyroid Under Observation	407	THYROID	6.568	2014	Japan
9	The changing incidence of thyroid cancer	396	NATURE REVIEWS ENDOCRINOLOGY	43.33	2016	USA
10	Papillary thyroid microcarcinoma: A study of 900 cases observed in a 60-year period	377	SURGERY	3.982	2008	USA

### Publication distribution among author analysis

In total, 4451 authors participated in PTMC-publications, with each one co-written by 4.05 authors on average. The top ten productive authors were from Asian research institutions, particularly South Korea (**Table 5**). According to Lotka law, they were regarded as the “core authors” in PTC research field. The top ten authors contributed 251 (22.86%) papers. The top three authors who published a high number of manuscripts were Miyauchi Akira (46, 4.19%), Ito Yasuhiro (44, 4.01%), and Kim Eun-Kyung (23, 2.09%). Kuma Hospital in Japan and Yonsei University in South Korea had most of the PTMC’s top researchers.

**Table 5:** Top 10 authors most frequently appearing in the publications.

		Author	Publications	TGCS	TLCS	Institution	Country
1		Miyauchi Akira	46	3348	1426	Kuma Hospital	Japan
2		Ito Yasuhiro	44	3224	1358	Kuma Hospital	Japan
3		Kim Eun-Kyung	23	1382	302	Yonsei University	Korea
4		Kwak Jin Young	22	1300	286	Yonsei University	Korea
5		Chung Woong Youn	20	862	192	Yonsei University	Korea
6		Shong Young Kee	20	791	297	Ulsan University	Korea
7		Zhang Ying	20	351	147	Chinese People's Liberation Army General Hospital	China
8		Kim Tae Yong	19	788	293	Ulsan University	Korea
9		Miya Akihiro	19	2233	1003	Kuma Hospital	Japan
10		Yoon Jung-Han	18	373	141	Chonnam National University	Korea

TGCS, total global citation score; TLCS, total local citation score.

### Co-occurrence analysis of keywords

**Figure 5:**
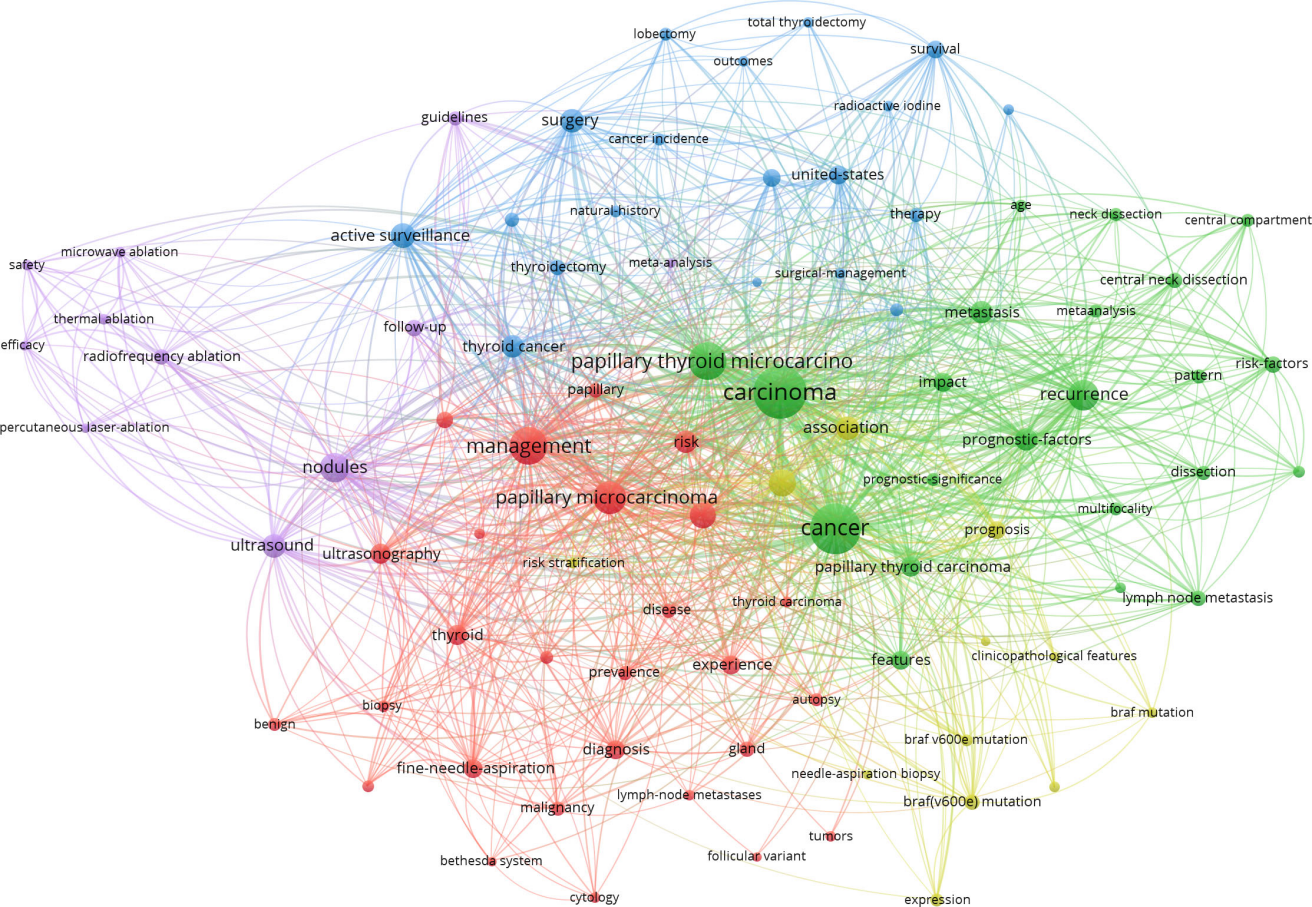
Clustering network of terms in PTMC publications from 2000 to 2021. The most common terms used in the scientific literature were investigated to identify relationships among the extracted terms. Dots represent keywords, and the higher the frequency of keywords, the larger the dots.

**Figure 6:**
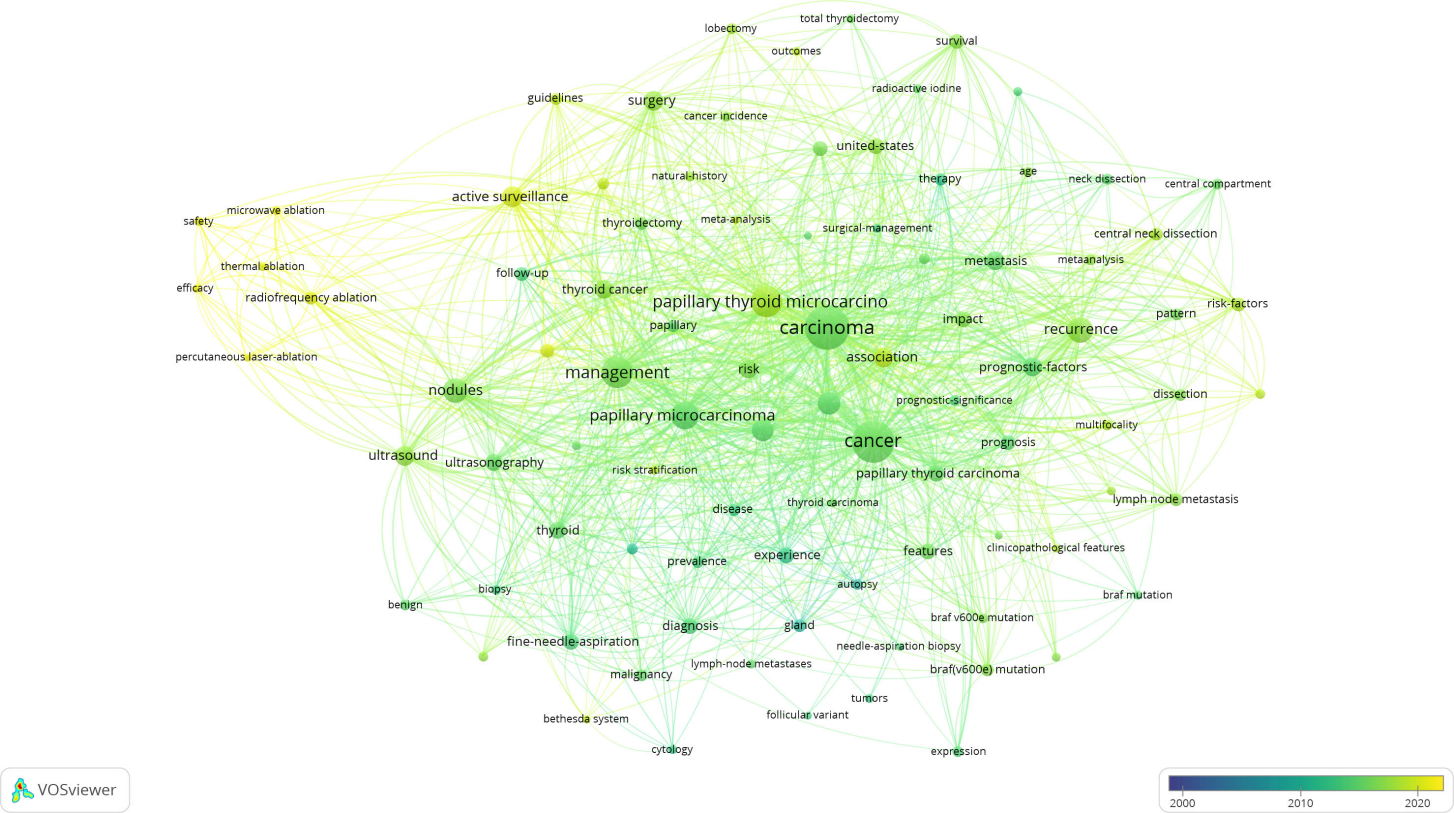
Overlay visualization of terms in PTMC publications from 2000 to 2021.

In co-occurrence analysis of keywords, 64 terms appeared more than or equal to 30-times and were classified into 5 clusters (**Figures 5**, **6**). Cluster 1 contained 21 keywords that indicated recurrent and prognostic factors of thyroid cancer; frequently mentioned keywords were carcinoma, recurrence, metastasis, prognostic factor, and lymph node metastasis. Cluster 2 included 16 keywords that referred to the management, ultrasound diagnosis, and treatment of micropapillary thyroid carcinoma. Cluster 3, relevant keywords included experience, prognosis, autopsy, and prevalence. Cluster 4, primary keywords included were active surveillance, surgery, increasing incidence, and progression. Cluster 5 pointed to BRAF and other gene expressions; the main keywords included BRAF (V600E) mutation and expression.

### Development and Frontier research

**Figure 7:**
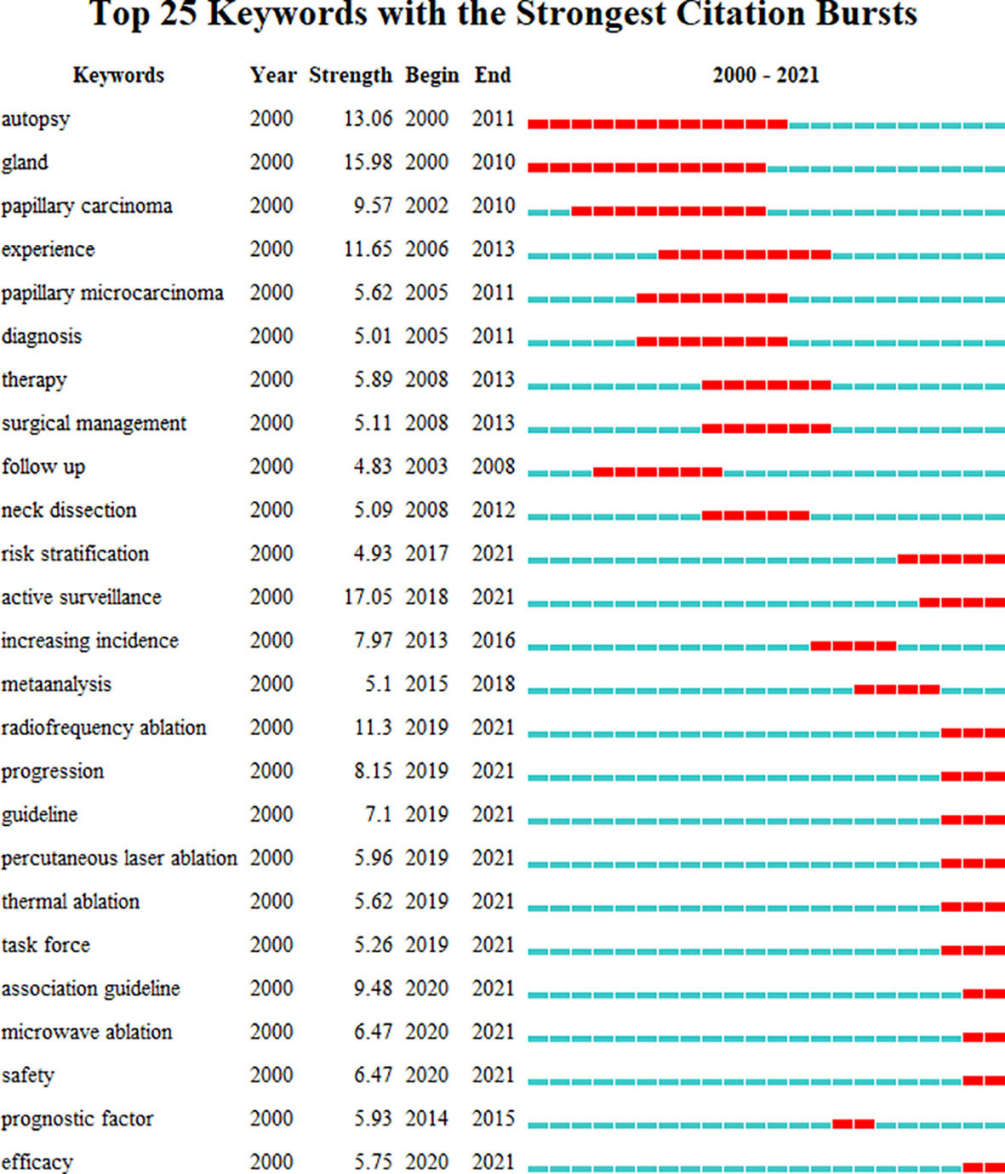
Top 25 keywords with the strongest citation bursts. Keywords with the strongest citation bursts in the scientific literature were analyzed and visualized in the keyword’s bursts map. Each short line represented a year and lines in red stood for the burst detection years. Keywords with red lines extend to the last year can indicate the research frontiers in a short period of time in the future.

Short keyword bursts were used as sensitive indicators to reflect current research trends. A keyword burst graph was generated using CiteSpace that shows the intensity of the burst and the year it started or ended (**Figure 7**). The following were the initial research directions: autopsy (began in 2000), gland (began in 2000), and papillary carcinoma (began in 2002), which later progressed to the investigation of various surgical treatment methods and diagnosis. Follow-up studies of patients in various research institutions gradually attracted attention towards the research on PTMC, such as experience (began in 2006), papillary microcarcinoma (began in 2005), diagnosis (began in 2005), therapy (began in 2008), surgical management (began in 2008), follow up (began in 2003), and neck dissection (began in 2008). Thyroid cancer screening was questioned (15). New treatments and concepts besides surgery emerged. This trend can be seen in research keywords, for example, risk stratification (began in 2017), active surveillance (began in 2018), increasing incidence (began in 2017), progression (began in 2019), guidelines (began in 2019), radiofrequency ablation (began in 2019), safety (began in 2020), prognostic factor (began in 2014), and efficacy (began in 2020).

## Discussion

This study used bibliometrics and visual analysis methods to discuss the research trends and hot spots in PTMC research field from 2000 to 2021. According to this study, PTMC research has shown an increasing trend over the last 20 years, with 2019 being the year with the most published literature.

During the 20 years, 1,098 papers from 75 countries were published. China published the most publications (329), but its h, g, and m indexes were lower than South Korea and the USA. Japan published 73, which was more than publications from South Korea and the USA. It is worth noting that South Korea accounted for five of the top listed research institutions in the number of publications, indicating that South Korea led PTMC research.

Keywords co-occurrence (**Figure 5**) obtained by VOSviewer 1.6.17 can be used to understand the primary research directions of a field. In this study, there were 2,380 keywords; 64 of them appeared more than 30 times. Cluster analysis classified the 64 terms into 5 clusters.

Cluster 1 was predominantly concerned with disease recurrence. The inconsistency in the risk of recurrence was primarily due to the heterogeneity of thyroid cancer characteristics, including surgical scope, pathological factors, and clinical factors. Therefore, different studies reported different risk factors for recurrence. Age, sex, tumor size, extrathyroidal extension (ETE), multifocality, and lymph node metastasis were all potential risk factors (16–18). First, for the more controversial unilateral PTMC, a recent study revealed that total thyroidectomy was associated with a lower recurrence rate with no significant difference in survival (19). In addition, prophylactic central neck dissection during surgery may reduce the recurrence rate (20). Second, compared to patients with unifocal PTC, multicentric PTC patients were older, had advanced TNM staging, and had a higher recurrence rate (21). Metastatic lymph node ratio higher than 0.44 was linked with an increased risk of locoregional recurrence, mostly in the lateral neck (22). However, tumors smaller than 5 mm were less likely to recur (23).

Cluster 2 was about the application of ultrasound. Ultrasound is one of the main diagnostic methods for thyroid cancer. The following ultrasonic features of thyroid nodules were significantly associated with PTMC: an irregular shape; an aspect ratio of 1; an unclear boundary; blurred margins; internal heterogeneous hypoechogenicity; and microcalcification (24). An ultrasound can diagnose thyroid cancer by fine-needle aspiration biopsy (FNAB). Lyu Yi-jun’s study presented that the malignancy rate of PTMC diagnosed by FNAB was highly consistent with the histopathological diagnosis (88.2% vs. 89.5%) (25). FNAB had 87% sensitivity to PTMC smaller than 5 mm (26). Third, ultrasound is an important method to predict lymph node metastasis (27). However, in Ito Y’s study, the specificity and sensitivity of ultrasound for central lymph node metastasis diagnosis were 99.1% and only 10.9%, respectively (28). Although ultrasound is now routinely revised after surgery, some studies believe that due to the very low recurrence rate of PTMC, monitoring *via* ultrasound one to two years after surgery may be unnecessary (29).

Cluster 3 was related to the prevalence of PTMC. The incidence of thyroid cancer in South Korea increased dramatically in 2000 and is now increasing by 25% per year. In 2011, approximately 40,568 people were diagnosed with thyroid cancer, and the incidence of thyroid cancer became15 times higher than in 1993 (30). According to Louise Davies’ research, medical imaging technology and diagnostic skills enabled the discovery of many asymptomatic cases of subclinical thyroid cancer (31). A study in Greece found an increased prevalence of PTMC (63.6% of all thyroid cancers) from 2007 to 2016 due to frequent use of FNAB (32). Positive diagnosis and treatment may also increase the patient’s distress, significantly affecting the patient’s subsequent quality of life.

Cluster 4 mainly correlated with active surveillance (AS). Due to the favorable prognosis of PTMC, it has a mortality rate of 0.3%, even in the presence of distance metastasis (33). Since the 1990s, two Japanese institutions, the Kuma Hospital and the Cancer Institute Hospital (CIH), have conducted prospective clinical studies of active surveillance (AS) in low-risk (T1aN0M0) PTMC. These studies show good results, low cancer growth rates, and lymph node metastasis with no deaths during AS (34–37). Active surveillance has been proposed as a first-line treatment for low-risk PTMC because tumors grow slowly, have low mortality and metastasis rate, and are safer and less expensive than surgery (38). However, AS is unsuitable for some PTMC patients, such as those with central lymph node metastasis (CLNM) (11). The disease progression of PTMC during active surveillance varied greatly according to age at onset. People in their 20s and 30s were more likely to develop disease progression than people over 50 (39). An Australian study found that surgery costs the same as 16.2 years of active surveillance (40). Moreover, AS was not recommended in the following conditions: lymph node metastasis, tumors involving or attached to the esophageal or recurrent laryngeal nerves, and age under 20 (41).

Cluster 5 was concerned about BRAF (V600E), which accounts for 60-80% of papillary thyroid carcinoma (PTC) and is a prognostic marker for risk stratification in PTC patients (42). Li Fei’s research suggested that BRAF mutations were associated with multifocal thyroid cancer, extrathyroidal extension, lymph node metastasis, and advanced stages regardless of age (43). However, a recent study by Kim Kwangsoon showed that BRAF mutation is not a risk factor for lateral neck lymph node metastasis (44). In addition to the BRAF gene, TERT promoter mutation has been reported as a newly discovered prognostic PTC gene mutation (45). However, the probability of TERT mutation is low (46). Liu et al. first reported a link between mutations of the TERT promoter and BRAF (V600E) in PTC, and subsequent studies further demonstrated the coexistence of TERT promoter and BRAF(V600E) mutations in thyroid cancers and their association with the most aggressive PTC clinical factors and worse prognosis (47–50).

The keyword co-occurrence (**Figure 7**) using CiteSpace 5.8R3 exhibited the trending topics in this field, while burst keywords represented the frontier topic. Burst keywords revealed the change of research hotspot. The recent burst of keywords, in particular, indicated underlying trends and possible frontiers in PTMC. It was seen that individual treatment with PTMC will be proposed more in the future, specifically “Risk stratification” and “radiofrequency ablation.”

Risk stratification is important for managing thyroid cancer. Patients with no family history of thyroid cancer or personal head and neck irradiation, no lymph node or distant metastasis at diagnosis, ETE, unifocal micro-PTC, no aggressive histological features (tall-cell, hobnail subtypes), and no vascular invasion were considered low-risk patients (51). Unfortunately, some of these data are available only after surgery. Tumor size is one of the recognized risk factors for thyroid cancer. In addition, there were many factors associated with poor prognosis, such as extensive fibrosis (52), age (more than 55 years) (53), tumor multifocality, and lymph node metastasis at presentation (54).

Thermal ablation (TA) has been widely used and regarded as a safe and effective treatment for low-risk PTMC. There have been many studies related to the safety of TA. In a retrospective clinical trial involving 311 patients, there was no difference in the recurrence rate and disease-free survival rate of low-risk PTMC between TA and lobectomy groups. However, there were significantly fewer patients with postoperative hypoparathyroidism than in the operation group (55). In addition, TA exhibited good efficacy in treating thyroid cancer lymph node metastasis, with a reduced rate of metastatic lymph nodes in 208 patients (412 lymph nodes) of 93%–94% (56). However, using TA in treating PTMC remains controversial, with many opposing voices in China. In a single-center study, 23 patients with thyroid cancer treated with TA underwent surgery again, and five had FNAB results inconsistent with postoperative pathology, demonstrating lymph node metastasis (57). In another retrospective study, 12 patients underwent surgery after TA treatment, with lymph node metastasis in 66.7% (8/12) of patients. Adhesion of post-ablation lesions with strap muscle was observed in six cases. Strap muscle was found to be cauterized in five cases, and notably, the recurrent laryngeal nerve was involved in one case (58). So, lymph node or distant metastasis before and after ablation in patients with primary PTMC is difficult to assess (59).

Guidelines and consensus differ slightly between associations regarding the scope of application of TA for PTMC. The American Thyroid Association Management Guidelines in 2015 and the South Korean Thyroid Radiofrequency Guidelines in 2017 recommend that TA should be employed only in high-risk surgery patients and those who refuse additional surgery (59, 60). The 2018 Austrian practice guidelines do not recommend TA in malignant thyroid nodules (61). In 2018, the Chinese expert consensus proposed the indications for PTMC treatment by TA, but only under very strict conditions. Non-pathological high-risk subtypes, tumor diameter ≤5 mm, no lymph nodes or distant transfer of evidence, and so on are examples of nine items that must be met simultaneously (62). The 2021 European Thyroid Association practice guidelines and the 2021 international multidisciplinary consensus statement suggest that TA should be considered for PTMC patients who are at risk of surgery, have a short life expectancy, have other medical conditions, are unwilling to have surgery, or want to stop AS (63, 64). The international mainstream association guidelines and statements still consider surgery the first choice for PTMC patients. TA is a relatively new technology that has emerged in recent years. After treatment, the follow-up time is insufficient, and the long-term safety and efficacy have not been evaluated.

## Limitations

Some deficiencies should be taken into account when interpreting the results of this study. First, the publications were derived solely from the Web of Science Core Collection databases. Second, only English-language publications were included in our analysis. Furthermore, new publications may subsequently be added to the December 2021 queue, but the database was not updated at the time of retrieval. However, this part of the literature was very small and will not cause a large error in the analysis. Despite these limitations, this study reveals the future research trends and hot spots in this field to some extent and uses bibliometrics to sort out this field for later researchers.

## Conclusions

To sum up, this study summarizes the global research trends of PTMC. Thyroid was the top productive journal. China has the largest number of publications, and the USA has the most citations. The University of Ulsan of Korea has done a great deal of research in this area and has contributed the most publications. The main research topics were the recurrence and metastasis of PTMC and the application of ultrasound in PTMC. Research frontiers were risk stratification of PTMC, active monitoring, and thermal ablation for PTMC. We believe these findings will provide useful information for researchers to explore PTMC further.

## Data availability statement

The raw data supporting the conclusions of this article will be made available by the authors, without undue reservation.

## Ethics statement

This study is a bibliometric analysis of the articles related to papillary thyroid microcarcinoma, and the data were obtained from the Clarivate Analytics Web of Science Core Collection database, so ethical approval and consent to participate were not necessary for this paper. Informed consent was obtained from all individual participants included in the study.

## Author contributions

This work was conceived by Hao Zhang and Zhongqing Wang. Data was collected and downloaded by Kefan Chen, Dalin Zhang, Ting Zhang and Liang He. The visualization work was performed by Kefan Chen and Wei Sun. The manuscript was written by Kefan Chen and Wei Sun. Hao Zhang, Zhongqing Wang, Dalin Zhang, Ting Zhang and Liang He helped to revise manuscript and proposed constructive opinions. All authors contributed to the article and approved the submitted version.

## Conflict of interest

The authors declare that the research was conducted in the absence of any commercial or financial relationships that could be construed as a potential conflict of interest.

## Publisher’s note

All claims expressed in this article are solely those of the authors and do not necessarily represent those of their affiliated organizations, or those of the publisher, the editors and the reviewers. Any product that may be evaluated in this article, or claim that may be made by its manufacturer, is not guaranteed or endorsed by the publisher.
